# Suppressive Effects of Intrathecal Application of Diazepam on Visceral Pain and Hyperalgesia Induced by Intracolonic Instillation of Formalin

**Published:** 2006-02

**Authors:** Jinghui Huang, Libing Liu, Yumei Zhou, Jun Yu, Jiao Deng

**Affiliations:** 1 *Cadet Brigade Squadron Twelve, Fourth Military Medical University, Xi’an 710032, China;*; 2 *Center of Teaching Lab, School of Basic Medicine, Fourth Military Medical University, Xi’an 710032, China*

**Keywords:** colonic inflammation, diazepam, formalin, hyperalgesia, visceral p

## Abstract

Using an animal model of colonic inflammation, the effects of intrathecal (i.t.) diazepam (0.02 mg/kg, 0.08 mg/kg and 0.15 mg/kg) on visceral pain-related behaviors and hyperalgesia associated with colonic inflammation were investigated. In this visceral pain model, acute visceral pain response was induced by intracolonic (i.c.) injection of 0.5 ml of dilute formalin (2%, 5% and 10%) in rats, and the peak pain behavioral response and hyperalgesia were evoked by i.c. 5% formalin. I.t. diazepam (0.02 mg/kg, 0.08 mg/kg and 0.15 mg/kg) followed 10 min later by i.c. injection of 5% formalin, attenuated the visceral pain behaviors induced by 5% formalin in a dose dependent manner. Of the three doses tested, the duration of the suppressive effect of 0.15 mg/kg diazepam on visceral pain was the longest, which is 60 min compared with 45 min at other two doses. Moreover, i.t. pretreatment with 0.08 mg/kg diazepam attenuated the hyperalgesia induced by i.c. injection of 5% formalin. The findings in our studies shown that i.t. diazepam had a suppressive effect on visceral pain associated with noxious stimulation of colon, and provided evidence that diazepam may be used as an analgesic drug in the future.

## INTRODUCTION

Visceral pain is the most common form of pain produced by disease, and one of the most frequent reasons why patients seek medical attention. Nonetheless, most of what we know about pain mechanisms is derived from studies of somatic rather than visceral nociception. However, the more we know about the mechanisms of somatic and visceral sensation, the more we realize that these two processes, while having many common features, also have important differences. For example, visceral pain is characterized by its referral to the body wall. In the zone of referral, patients also report tenderness known as referred visceral hyperalgesia.

γ-Aminobutyric acid (GABA) is the predominant inhibitory neurotransmitter in virtually every area of the central nervous system (CNS), and it activates GABAA receptors to open chloride ion channels and mediates fast inhibitory synaptic transmission. GABAA receptors belong to the family of homologous pentameric transmitter-gated ion channels and many classes of drugs interact with them (11). Among these drugs are the positive allosteric modulators acting at the benzodiazepine binding site, which is an integral component of this pentameric chloride-selective pore complex (20). Diazepam, a benzodiazepine receptor agonist, has been shown to act as a positive allosteric modulator to enhance GABA-mediated Cl- conductance in cultured/dissociated central neurons and brain tissue slices (2, 3, 19). Potentiation of GABAA receptor mediated synaptic inhibition is believed to contribute to the anxiolytic, anticonvulsant, and sedative effects of diazepam.

The rat sacral dorsal commissural nucleus (SDCN), which represents the dorsal gray matter of the central canal in the lower lumbar and sacral spinal cord, serves as a relay of sensory information from the pelvic viscera. Moreover, previous studies have revealed that GABA-like immunoreactive neurons and terminals and GABA receptors are densely located in the SDCN (5). All these evidences indicated that modulation of GABAA receptors by benzodiazepines has the potential for significant influence on spinal nociception. In addition, there are reports that intrathecal (i.t.) diazepam suppresses somatic sensation in pentobarbital-anesthetized rats (18). However, little information about the suppressive effect of diazepam on visceral pain was reported until now. So, in this study, on the basis of a visceral-specific behavioral model developed by intracolonic (i.c.) instillation of formalin, the possible effects of diazepam on visceral pain-related behaviors and hyperalgesia associated with colonic inflammation were investigated. It is hoped that information obtained will lead to clinical implications that diazepam have a suppressive effect on visceral pain when it’s used as anxiolytics and anticonvulsants.

## MATERIALS AND METHODS

### Animals

Adult male Sprague-Dawley albino rats weighing from 180 to 220 g were provided by Laboratory Animal Center of the Fourth Military Medical University (FMMU) and use of the animals was reviewed and approved by the Institutional Ethical Committee of the FMMU. The IASP's guidelines for pain research in animals were followed. The animals were housed in plastic boxes in group of 3 with food and water available ad libitum in a colony room with controlled temperature (242°C), humidity (50%∼60%), and a 12:12 h light-dark cycle. The rats were acclimatized to the laboratory and habituated to the observation chamber for at least 30 min each day for 5 days before testing.

### Spinal catheterization procedure

For i.t. administration of the drugs, chronic i.t. catheterization was modified according to previous reports (22, 23). Briefly, a PE-8 plastic tube (inter diameter=0.28 mm; out diameter=0.60 mm, Natume, Tokyo, Japan) was inserted from the T2-T3 level into the sub-arachnoid space of the rostral lumbosacral enlargement under continued intraperitoneal pentobarbital sodium anesthesia (40 mg/kg). Experiments were performed at least 5 days after the operation to minimize any influence of the catheterization procedure. Only those without motor disturbances or other abnormal signs were used. After tests, each rat was checked for the placement of the end of the catheter, those with incorrect placement or local pathological changes were not used for experimentation.

### Nociceptive behavior assessment

A 30 cm × 30 cm × 30 cm transparent observation chamber with a transparent glass floor was placed on a supporting frame of 45 cm high above the experimental table. Before starting the session, the rat was placed in the observation chamber for 30 min to accommodate to its new environment. Petroleum jelly (Vaseline) was then applied in the perianal area to avoid the stimulation of somatic areas by contact with the irritant chemicals. Then the animal was anesthetized with a small amount of diethyl ether, while 0.5 ml of solution (saline, 2% formalin, 5% formalin or 10% formalin) was administered by introducing a fine cannula with a rounded tip (inter diameter=1.1 mm; out diameter=1.5 mm, 8 cm long) into the colon via the anus. The rat was replaced in its observation chamber. The spontaneous behaviors were observed for 90 min, and the latency of the first such behavior was also noted. All selected behaviors related to visceral nociception (i.e., licking or nibbling abdomen or perineal area, body stretching, contraction of the flanks, and whole body contraction, were listed here in increasing order of nociceptive intensity. The latter behavior was further graded according to the duration of the given episode: for less than 30 s, between 30 s and 1 min, and for more than 1 min. The nociceptive response was then calculated for each of the successive 15 min periods, using the formula (17). In all experiments, animal behaviors were recorded blind as regarded control or treated status.

### Examination of mechanical hyperalgesia

In the rats with i.c. saline and 5% formalin, the frequencies of withdrawal responses to von Frey hairs of the abdomen, the hind paws and the tails were examined as tests of referred hyperalgesia immediately after behavioral tests. Five hairs with forces of 47, 60, 80, 115, 156 mN were applied 10 times each in ascending order of force, and the number and intensity of responses noted. The forces exerted by the von Frey hairs were checked before testing using a micromanipulator to advance them towards a sensitive balance. The hair was applied for 1∼2 s, with an inter-stimulus interval of 5∼10 s. Care was taken not to stimulate the same point twice in succession, to avoid ‘wind-up’ effects or desensitization. The appearance of a sharp withdrawal of the stimulated region on the application of a hair was considered as a withdrawal response. In addition, immediate licking or scratching the stimulated site and jumping were also regarded as withdrawal responses for abdomen (12).

### Assessment of colonic inflammation

At the end of behavioral test, one rat with i.c. saline and one with i.c. 5% formalin were chosen randomly, and they were killed by an intraperitoneal pentobarbital overdose. The colon was excised and opened longitudinally. Histological assessment was then performed. The tissue was removed, fixed overnight by immersion in Bouin’s solution, dehydrated, embedded in paraffin and stained with haematoxylin and eosin.

### Effects of i.t. diazepam on visceral pain-related behaviors and hyperalgesia induced by i.c. 5% formalin

Four groups of rats (n=8 per group) were intrathecally pre-treated with 10 μL of diazepam at three doses (0.02 mg/kg, 0.08 mg/kg and 0.15 mg/kg) and with saline vehicle 10 min before i.c. 5% formalin to examine the effects of i.t. diazepam on

### Visceral pain–related behaviors

In the rats intrathecally pretreated with 10 μL of saline and diazepam (0.08mg/kg), the responses to mechanical stimulation of the abdomen, the tail and the hind paws were tested with von Frey hairs as described above.

### Statistical analysis

The SPSS software was used to determine the statistical significance of the differences. The results were expressed as means ± S.E.M. of the value of pain scored per 15 min, of the latency of the first visceral pain behavior and of the response frequency at each von Frey hair. Group differences were considered statistically significant at *P*<0.05 using a repeated-measures analysis of variance for visceral pain-related behaviors recorded along, test for the response frequency at each intensity of von Frey hair, and test for the latency of the first visceral pain behavior.

## RESULTS

### Formalin induced inflammation in the colon

**Figure 1:**
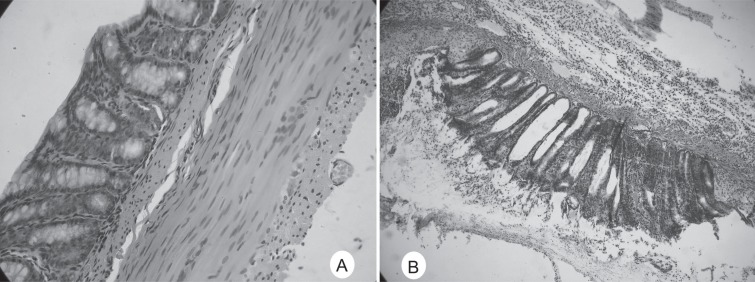
Colonic inflammation induced by i.c. 5% formalin.

Gross inspection of the colon after i.c. saline showed an apparently normal colon as shown in Fig. 1A. However, I.c. 5% formalin evoked plasma extravasation in the colon that was significantly different from that observed after saline administration. As shown in Fig. 1B, The architecture of the mucosa was disorganized and the loss of crypt epithelial cells can be seen. Mild cellular infiltration in lamina propria is also noted.

### Nociceptive responses after i.c. injection of formalin and saline

**Figure 2:**
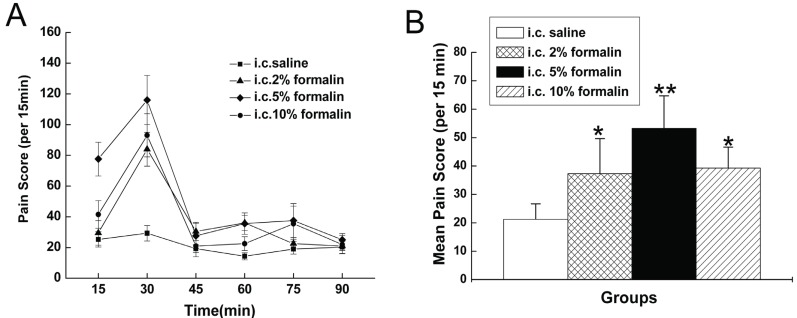
Visceral pain responses induced by formalin. (A) Curve graph shows the mean time courses for 90 min after i.c. formalin. (B) Column graph shows the mean numbers of the pain scores per 15 min averaged from 6 time blocks of 90 min after i.c. formalin. *P<0.01; **P<0.001vs rats with i.c. saline.

I.c. saline was followed by the appearance of a small number of abdominal licking behaviors, while i.c. formalin produced a tonic, mono-phasic nociceptive response lasted for about 75 min, which is significantly different from that observed in saline-treated rats (Fig. 2). The number of behaviors evoked by formalin was concentration-dependent, with more behavioral responses evoked by higher concentrations, but 5% formalin evoked more visceral pain-related behaviors than 2% and 10% formalin (Fig. 2B). Moreover, there was also evidence of a ‘ceiling effect’ with the highest concentration tested. During the 90 min test period, there was a maximal visceral pain response at 15 min-30 min at all concentrations tested, then the number of visceral pain-related behaviors decreased to baseline level at 75 min after formalin injection.

**Figure 3:**
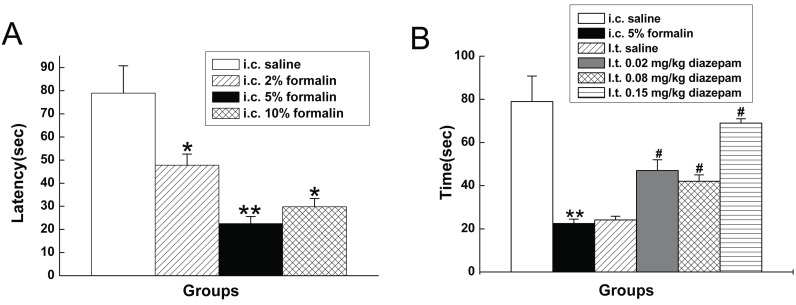
Latency of the first visceral pain-related behavior. *P<0.05, **P<0.01 vs rats with i.c. saline. #*P*<0.05 vs rats with i.c. 5% formalin.

The latency of the response to administration of saline was greater than 40 s in all rats tested. In contrast, the latency was much shorter when i.c. administrated of formalin. As shown in Fig. 3A, the latency of the response to administration of saline, 2% formalin, 5% formalin, 10% formalin were 79.1 ± 11.8 s, 47.8 ± 4.8 s, 22.5 ± 1.4 s, 29.8 ± 2.5 s.

### Effect of i.t. diazepam on spontaneous behaviors evoked by i.c. 5% formalin

**Figure 4:**
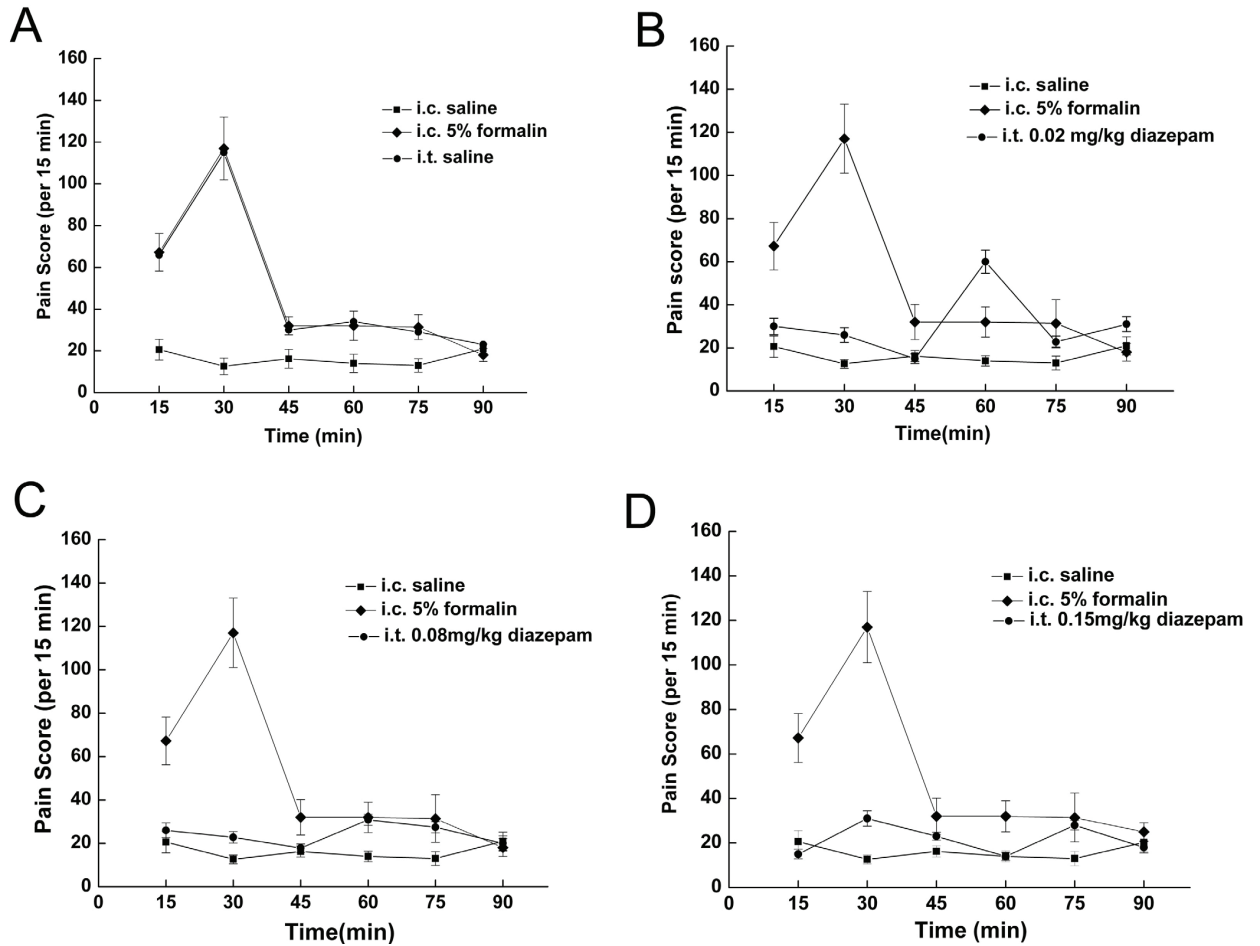
Effect of i.t. diazepam on visceral pain-related behaviors induced by i.c. 5% formalin.

**Figure 5:**
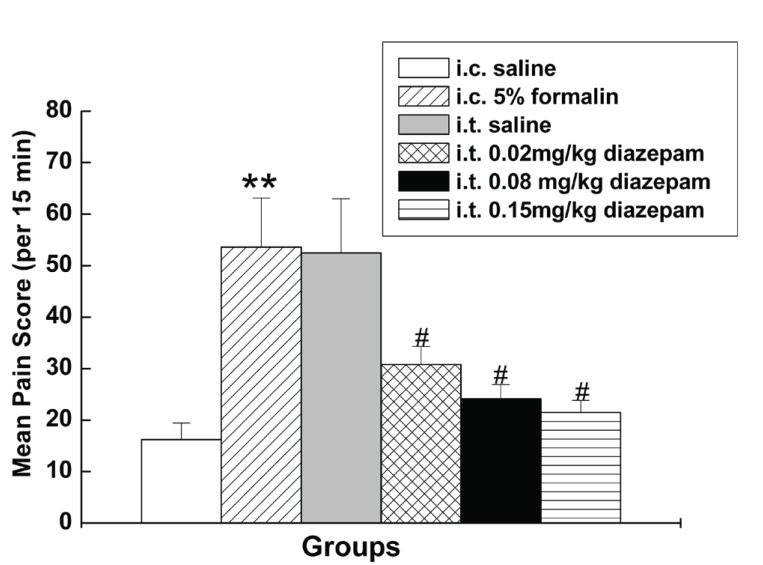
Effect of i.t. diazepam on visceral pain-related behaviors induced by i.c. 5% formalin. **P<0.001 vs rats with i.c. saline; #*P*<0.001 vs rats with i.c. 5% formalin.

I.t. saline had little influence on visceral pain-related behaviors induced by 5% formalin, while i.t. diazepam dose-dependently reduced the number of visceral pain-related behaviors induced by i.c. 5% formalin treatment (Fig. 5), with higher dose intrathecally administrated, to more extent the number of behaviors was reduced. As shown in Fig. 4, the duration of the suppressive effect of i.t. diazepam on visceral pain behaviors lasted for 45 min-60 min after i.c. administration during the 90 min test period, which was also dose dependent, with 60 min at 0.15 mg/kg, and 45 min at other two doses (0.02 mg/kg, 0.08 mg/kg).

The latency of the response to i.c. 5% formalin treatment in the rats with i.t. diazepam was much longer than that observed in the rats without i.t. diazepam. As shown in Fig. 3B, the latency of the response to i.c. 5% formalin in the rats with i.t. saline and i.t. diazepam (0.02 mg/kg, 0.08 mg/kg and 0.15 mg/kg) were 24.1 ± 1.7 s, 47.3 ± 5.3 s, 42.3 ± 3.5 s, 69.2 ± 7.2 s, respectively.

### Withdrawal responses to mechanical stimulations

**Figure 6:**
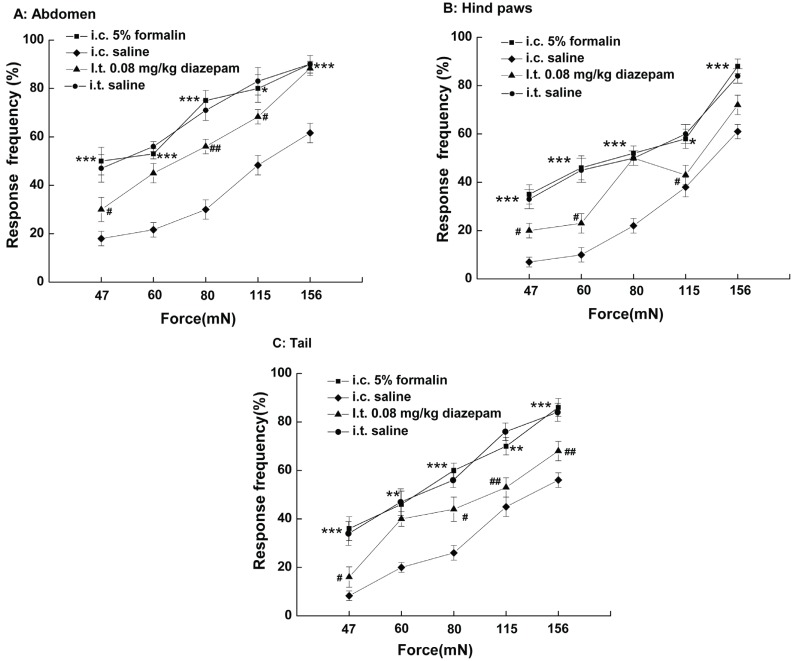
Responses to mechanical stimulation of different body areas with von Frey hairs of five intensities. *P<0.05; **P<0.01, ***P<0.001 vs rats with i.c. saline. #*P*<0.05, ##*P*<0.01vs rats with i.c. 5% formalin.

The tail, the hind paws and the abdomen were similar sensitive when tested their responsiveness to von Frey hairs 90 min after i.c. saline. However, 90 min after i.c. 5% formalin treatment, there was a marked increase in responsiveness in all three sites tested, such that the stimulus response curve shifted to the left (Fig. 6), which was called hyperalgesia. This phenomenon was attenuated by i.t. diazepam to some extent. As shown in Fig. 6, Rats with i.t. diazepam (0.08 mg/kg) were less responsive to von Frey hairs than the ones without i.t. diazepam 90 min post i.c. 5% formalin treatment, with a pronounced shift of stimulus-response curve to the right (Fig. 6).

## DISCUSSIONS

Abdominal licking has been described as a common response in practically all models of cystitis, ureteric calculosis, uterine inflammation and stimulation of colon (8-10, 17, 26). However, abdominal reaction (body stretching, contraction of the flanks and whole body contraction) seems to be more specifically associated with noxious stimulation of colon in both rats and mice (17) although it has also been reported in some studies of cystitis in rats (10). So, abdominal licking and abdominal reaction were chosen as indexes of visceral pain in this visceral pain model, and in our studies, i.c. formalin produced a tonic, mono-phasic nociceptive response which is different from biphasic nociceptive response induced by colonic wall injection of formalin (17). Moreover, ‘ceiling effect’ noted with the higher concentration of formalin tested in our experiment, may be a behavioral phenomenon in which the active expression of pain-related behaviors are partially occluded by freezing reaction. Alternatively, it may be due to a local desensitization resulting from mucosal damage, as has been described for relax responses to colon distension after mucosal inflammation (6).

Benzodiazepines exert their effect by binding to a specific site in the macromolecular complex of the GABAA receptor, increasing their affinity for GABA. This results in an increased opening frequency of these ligand-gated Cl- channels, thus potentiating the effect of GABA release in terms of inhibitory effects on the postsynaptic cells (21). Several studies indicated i.t. diazepam suppressed nociceptive reflexes in pentobarbital-anesthetized rats and the nociceptive activity of convergent neurons in the spinal cord during ischemia and reperfusion of their receptive fields on the rat's tail, both of which were related with somatic sensation (7, 18). Our present results confirmed the antinociceptive effects of diazepam in terms of visceral sensation using a model of inflammatory colonic pain. However, the exact mechanisms of the inhibitory effect of i.t. diazepam on visceral pain were still unclear, but recent evidences suggest that the interaction between diazepam and GABAA receptor complex in the SDCN might be one of the underlying mechanisms.

SDCN is a cell column located in the dorsal gray matter of central canal in the lower lumbar and sacral spinal cord (13). It receives primary afferent inputs from both pelvic and pudendal nerves, which provide major innervation of pelvic organ (14, 24). Accordingly, the SDCN serves as a relay of sensory information from the pelvic viscera. An important role of the nucleus is in nociception, as suggested by the increased expression of c-fos in the SDCN following both noxious chemical stimulation of the bladder and high-intensity electrical stimulation of the pelvic nerve (4, 13). The opinion that the projections from the SDCN participate in nociception was also indicated by clinical evidence, that discrete lesions of the dorsal columns relieved pelvic pain in patients (28). Axons of neurons in SDCN have been shown to travel in the dorsal columns to the gracile nucleus and in the ventrolateral quadrant to reticular formation (1, 25). Furthermore, neurons in this region receive descending inputs from several regions of brain known to modulate the spinal processing of nociceptive signals (15, 27). These characteristics thus make the SDCN an ideal place to study the transmission and modulation of the pelvic visceral nociceptive signals. Moreover, previous studies have revealed that GABA-like immunoreactive neurons and terminals and GABA receptors are densely located in the SDCN (3, 5). In our studies, diazepam was injected into the level of lower lumbar and sacral spinal cord, and the diazepam might exert its suppressive effect on visceral pain by potentiating the inhibitory effects of GABA through facilitating the uptake of Cl- into GABAergic neurons in SDCN, and it might be possible that in such a way the transmission of visceral pain information form periphery to the higher centers of the brain was partly inhibited. However, the effect of diazepam in the SDCN might be just one possible reason count for its suppressive effect on visceral pain. To discover the exact mechanisms of the analgesic action of i.t. diazepam, more works need to be done in the future.

Visceral pain differs from somatic pain in several clinical and physiological aspects and in particular, pain is often perceived by the sufferer as arising from somatic sites distant from the visceral injury (16). This phenomenon was called visceral-somatic hyperalgesia, which has been qualified using electrical and nature stimulation in patients with a variety of different visceral pain states, and has also been measured in animal models of visceral pain. It has been documented that in rats with an experimental ureteric calculosis a reduced threshold to electrical stimulation of the flank muscles was observed (10). What’s more, rats with uterine inflammation show increased responsiveness to palpation of the lateral flank muscles (26). In our studies, rats with colonic inflammation are more sensitive to mechanical stimulation in the abdomen, the hind paws and the tail. These sites are commonly innervated by dermatomes which share the same spinal innervation as the injured viscera, which has been previously reported in an animal model of mouse colonic inflammation (12) our studies shown that the referred punctuate mechanical hyperalgesia was attenuated by i.t. diazepam. This provides supporting evidence for the possibility that diazepam may be effective in preventing pain associated with inflammatory conditions of the colon. These findings may be of particular clinical importance since, in chronic visceral hyperalgesia syndromes, referred hyperalgesia can be a cause of suffering long after the immediate consequences of the primary insult has been resolved.

In conclusion, the results have shown that intrathecal diazepam is effective in attenuating visceral pain-related behaviors and referred hyperalgesia in an animal model of colonic inflammation. However, there are a lot of problems to solve before we use diazepam as an intrathecal drug to relieve visceral pain in clinical settings. When diazepam is used intrathecally in humans, whether there are adverse or side effects? Are there any other therapeutic functions? And what is the appropriate dose to relieve visceral pain. To answer all these questions, further studies are needed to be carried out.
